# PD30 - Management of pediatric anaphylaxis - comparison between a district general hospital (DGH) and a regional centre in UK

**DOI:** 10.1186/2045-7022-4-S1-P30

**Published:** 2014-02-28

**Authors:** Srinivas Jyothi, Georgina Harlow, Anand Kanani, Manju Kannath, Arun Ghose, Julia Richardson, Nicola Dowling, Nick Makwana

**Affiliations:** 1 Birmingham Children’s Hospital, Birmingham, United Kingdom; 2 City and Sandwell Hospital, Birmingham, United Kingdom

## Background

Anaphylaxis is a serious, life-threatening hypersensitivity reaction. The incidence of anaphylaxis is 4-5 per 100,000 persons per year and is reported to be increasing in recent years.

## Aims

We analysed management of suspected anaphylaxis in children at a DGH and a regional referral center in UK.

## Methods

A retrospective case note analysis was carried out between January 2007 and September 2012, which was compared to NICE (National Institute of Clinical Excellence) guidelines.

## Results

We identified a total of 81 cases from the DGH of which 71 case notes were analysed and a total of 30 cases from the regional centre.

**Table 1:** Initial management

Initial Management	Percentage of children who received intervention (%)	
	**DGH**	**Regional centre**

Adrenaline IM(pre-hospital + in hospital)	66(33; 33)	70(16:54)

Antihistamines	89	60

Steroids	87	46

Oxygen	37	33

Fluids	17	10

Nebulised salbutamol	76	40

**Table 2:** Compliance with NICE guidelines on discharge

On discharge	Percentage of children (%)	
	**DGH**	**Regional centre**

Allergy clinic follow up planned	92	54

Issued with adrenaline auto injector	69	10

Documented training in auto injector use if given	73	13

Patients receiving discharge information about anaphylaxis	73	23

Patients receiving discharge information fulfilling the criteria stated by NICE	0	0

Both centers’ were good at documenting acute clinical features (>95%) and the circumstances prior to symptom onset (>93%). Both hospitals need to improve their documentation of time of onset of reaction (50:30%), informing about biphasic reaction (8.5- 1%) and supply information regarding support groups (1.4-0%). Our study revealed no child received full discharge information according to NICE criteria.

The DGH performed better than the tertiary center in referral to specialist allergy services providing adrenaline auto injector and demonstration of auto injector.

## Conclusions

The DGH outperformed the tertiary center likely due to availability of specialist allergy services. We endeavor to improve our management by establishment of specialist allergy services at the tertiary hospital and anaphylaxis education among all doctors.

